# The role of environmental diplomacy and economic factors on environmental degradation

**DOI:** 10.1016/j.heliyon.2024.e24642

**Published:** 2024-01-17

**Authors:** Mohammad Maruf Hasan, Su Nan, Muhammad Rizwanullah

**Affiliations:** a School of International Studies, Sichuan University, Sichuan, 610065, China; b School of Economics, Sichuan University, Sichuan, 610065, China; c China Center for South Asian Studies, Sichuan University, Sichuan, 610065, China; d School of Public Administration, Xiangtan University, 411105, China; e South Asia Research Centre Xiangtan University, 411105, China

**Keywords:** GDP, GDP square, FDI, Environmental diplomacy, Environmental diplomacy square and renewable energy consumption, Environmental degradation and Kuznets curve theory

## Abstract

In recent years, rising carbon dioxide (CO2) emissions and other negative environmental effects of human activity have raised concerns about the planet's future. Rapid industrialization, urbanization, and economic activity have shown a surge in CO2 emissions, contributing to global warming and climate change. The main purpose of this study is to examine the impact of such as gross domestic product, gross domestic product square, foreign direct investment, environmental diplomacy, environmental diplomacy security, and renewable energy consumption on environmental degradation. This study provides a new perspective on environmental diplomacy in OECD countries using panel data econometric methodologies from 1991 to 2020. It contributes to our understanding of the role of environmental and economic factors in reducing CO2 emissions. The panel data is also analyzed by CD, CIPS, FMOLS, DOLS, and PMG-ARDL tests. However, as per the findings of this research, all the factors significantly impact environmental degradation (Co2 emission). Finding data to either confirm or deny the efficacy of the Environmental Kuznets Curve theory within the framework of OECD countries is possible through this approach. This policy framework attempts to solve the issues at the connection of environmental diplomacy and economic concerns by emphasizing cooperation and sustainability and incorporating environmental considerations into economic decision-making processes in OECD countries.

## 1 Introduction

There has been a growing awareness of environmental issues in the twenty-first century, and most individuals recognize serious environmental challenges [1]. Governments, companies, and civil society organizations unite to discuss the environmental challenges and come up with ways to persuade people to change their mindset so that we can address the planet's most pressing problems [2,3].

World leaders convene annually at conferences to ratify climate action treaties [4]. According to the United Nations Environmental Program (UNEP), countries have signed over 500 internationally recognized agreements over the past 50 years [5]. Because of the perceived global warming, some view these treaties as an example of environmental diplomacy that has yet to materialize. [5,6], while signatories emphasize their governments' commitment to a healthy environment. For instance, each year, the proportion of global CO2 emissions increases. Emissions peaked in 2014 at 360 million tons, up from 330 million in 1980. As a result, we wish to examine whether or not the amount of environmental diplomacy among countries affects environmental degradation. Diplomacy between countries to address environmental concerns [7] includes negotiation, persuasion, communication, and agreements. Countries sign bilateral, multilateral, and global treaties to demonstrate their dedication to mediation. The research that has already been done on globalization and environmental damage shows that environmental treaties have a direct effect on the amount of CO2 released, even when economic globalization is taken into account [[8], [9]]. For instance, states signatories to multiple environmental pacts will not ignore their domestic legislation in favor of international obligations under environmental diplomacy [10]. Thus, a government cannot relax its regulation of FDI as much as it would want because of its diplomatic obligations to other countries regarding environmental protection [11]. Globalization's impact on global climate change needs to be better understood due to a lack of research considering the relationship between environmental diplomacy and environmental degradation. Therefore, environmental diplomacy has yet to be considered in previous analyses of the causes of environmental degradation [12].

Therefore, environmental diplomacy is vital to preventing environmental degradation due to the lack of borders. The most effective approach to addressing the associated concerns is engaging in international discussions and establishing conventions. Therefore, countries consistently and indefinitely endorse environmental agreements to demonstrate their dedication to mitigating environmental degradation [13]. Furthermore, global initiatives, such as international treaties, provide a well-defined framework for addressing the immediate issues caused by climate change. Therefore, countries with diplomatic solid ties are expected to have lower levels of environmental degradation due to their commitment to treaties [14]. Conversely, governments with little diplomatic involvement are likely to experience higher levels of environmental degradation due to their incapacity to participate in such treaties [15].

Our research employs two distinct methodologies. It is the initial study, to start with to experimentally investigate the link between environmental degradation (CO2 emissions) and environmental diplomacy using variables from the OECD nations (for example, environmental security, renewable energy consumption, natural capital, GDP, and GDP square). Furthermore, our research offers significant perspectives on how diplomatic initiatives and economic metrics might facilitate environmental protection. Policymakers in OECD member nations will benefit from these results as they craft evidence-based policies that support sustainable development and lower CO2 emissions. The following sections comprise the organized format of the paper: Literature Review in section 2. The study strategy is explained in Section 3. The results and interpretations are presented in Section 4. The major findings are discussed in Section 5. Section 6 brings the paper to a close, while Section 7 describes the policy implications.

### 1.1 Objectives

The objectives are as follows:

1. To investigate the impact of environmental factors on environmental degradation.
2. To determine the impact of economic factors on environmental degradation.

## 2 Literature review

This literature review explores the relationship between economic indicators and environmental degradation, focusing on GDP, GDP square, FDI, Environmental Diplomacy, and Renewable Energy Consumption. It examines the effect of these variables, particularly on Co2 emissions, on global environmental health. The review emphasizes the importance of understanding these interconnected elements for developing effective strategies to address environmental challenges, foster economic growth, and promote international cooperation.

### 2.1 Theoretical framework

#### 2.1.1 Environmental Kuznets Curve theory (EKC)

The Environmental Kuznets Curve theory proposes that during the initial stages of economic development, environmental degradation increases due to industrialization and economic growth [16]. However, once a certain income threshold is reached, environmental degradation starts to decline as societies become wealthier and invest in cleaner technologies and environmental protection measures. This theory offers a framework to comprehend the relationship between GDP, GDP squared, and CO2 emissions in OECD countries [17]. The curve theory suggests that CO2 emissions also increase as GDP increases but at a decreasing rate. This means that as countries reach higher levels of economic development, they are more likely to adopt sustainable practices and decrease their carbon footprint [18]. Nevertheless, it is important to note that the relationship between GDP, GDP squared, and CO2 emissions may vary across different regions and contexts due to policy effectiveness and resource availability [19].

#### 2.1.2 International relation theory

Realist theories can be used to explain the motivations and behavior of states in environmental diplomacy. States prioritize their self-interest and national security in international negotiations, affecting environmental agreement outcomes [20]. On the other hand, constructivism can help us analyze how shared norms, ideas, and international institutions affect the effectiveness of environmental diplomacy and cooperation among OECD member countries. This theory emphasizes the role of social interactions and the power of ideas in shaping state behavior. It implies that perceptions of shared norms and the presence of international institutions that encourage cooperation affect states' willingness to cooperate on environmental issues. By applying constructivist analysis, we can gain insights into how these factors impact the success or failure of environmental diplomacy within the OECD [21].

### 2.2 Empirical evidence

It emphasizes how environmental issues and economic growth are related. Empirical research on CO2 has been influenced by the EKC model developed in the 1990. The study concludes in Section 6, and Section 7 discusses the consequences of policy. [19,20]. Grossman & Krueger (1995) were the first to use EKC to investigate empirically how the environment affects global trade. Three categories of influence are covered: structure, technology, and scale, which refer to how much the environment has changed due to technology. After careful consideration, the writers conclude that economic growth is the primary force forming our world [ [20,21]]. Academics integrate income, international trade, and FDI subsequently Grossman and Krueger (1995) to test the Kuznets hypothesis [22]. [23] Evaluate many factors and do a regression analysis on Gross domestic product per capita. The researchers confirm the validity of the pool of local firms held by EKC. In the worldwide sample, EKC rises with income. The hypothesis of EKC is used to explain that trade openness has an impact on environmental [[22], [24], [25]]. The long-term balance between consumption of energy and global trade and carbon dioxide emissions is shown by long-run computation using the Johansen cointegration approach on data from Pakistan, supporting the Kuznets hypothesis in that country. However, short-term estimates support neither EKC nor global trade because they need to find a correlation between carbon emissions and global trade [26]. Provide supporting evidence by stating that keeping EKC in South Africa is possible thanks to reduced energy pollution and trade openness. Furthermore, whereas [27] indicate a long term negative equilibrium between income and CO2 emissions in developed economies; however, the opposite is seen in rising economies. A positive association between rising levels of trade and prosperity and carbon emissions in the industrialized world was shown by Granger causality testing. However, for emerging countries, the fundamental relationship changes from carbon emissions to money and trade [28]. Found that carbon emission trading and information sharing reduced Co2 emission in China's medium and small markets. Other research looks at how globalization affects CO2 emissions. Only information on globalization's economic, social, and political aspects is present in Ref. [11] data set. Using the ARDL limits test [[13], [29]], find that globalization increases CO2 emissions in China. CO2 emissions in China have risen due to all three aspects of globalization. According to Ref. [15], freer trade and more economic integration have led to higher CO2 emissions. However [30], show no association between globalization and carbon emission in South Africa region, suggesting a neutral outcome. Using panel data from 25 industrialized nations throughout Western Europe, Asia, Oceania and North America [31] found no positive or negative effects of globalization on environmental health. Using a geographical regression model [32], demonstrates that rising economic globalization boosts CO2 emissions in 83 developed and developing nations. From 1990 to 2013 [12], analyses the trade-carbon connection for 102 nations. The researcher affirmed that exports and imports are mutually supportive, with exports reducing carbon emissions and raising imports. [9], came to a similar conclusion, finding that both long- and short-run export and import estimators lower carbon emissions. However, the effects of these adjustments are not seen until the fourth year. Foreign direct investment and carbon dioxide emissions have contradictory results. If there is no functional institutional structure, for instance, emancipation in financial institutions might lead to a rise in CO2 emissions, as [12] reported. However [33], Investigates how the BRICS countries' generation of renewable and non-renewable power is affected by foreign aid and energy aid inflows from 1995 to 2015. Results show that economic growth, environmental diplomacy, and foreign FDI condense Co2 emissions. The study suggests that policymakers should encourage increased investments in renewable electricity production for a sustainable future. A research conduct in China's regional districts [34] shows that inward and outbound FDI helps reduce carbon emissions. [35], investigated whether the growth of the capital market system and the influx of foreign funds may appreciate the utilization of RE. The researchers confirms that the capital growth markets and the inflow of FDI can boost RE projects and lead to CO2 emissions. To determine whether or not foreign capital inflow and energy consumption impact carbon dioxide emissions [36], used a pooled mean group (PMG). According to the authors, only when renewable energy consumption rises does FDI contribute to environmental sustainability. According to Ref. [37] findings, ASEAN nations' carbon emissions rise while receiving foreign investment, whereas they fall when increasing their energy efficiency and per capita income. [38], analyzed the effect of foreign direct investment on carbon dioxide emissions across 28 sectors of China's manufacturing industry between 2002 and 2015. They discovered that increased foreign direct investment (FDI) led to decreased carbon emissions. [39] studied how FDI entering and leaving an economy affected carbon emissions. They applied OLS regression estimators, fixed and random effects, and the generalized method of moments (GMM) to 115 economies, 34 of which were in Asia. [40], examined a connection between FDI, and CO2 emissions in Turkey but came up empty. The study also found that international trade affects carbon output. [41], evaluate the impact of foreign investment and new knowledge on China's industrial CO2 emissions. According to the research [42], R&D slows this trend while foreign investment accelerates industrial CO2 emission increases. The research shows that by 2030, FDI will drop by more than 5 % a year, whereas R&D will rise at a steady clip of more than 5 % yearly. Another recent study by Ref. [43], came to the same conclusion: more FDI means more carbon dioxide emissions. The current research also discusses the correlation between real GDP expansion and greenhouse gas emissions. For instance Ref. [44], examine the association among the gross domestic product and metropolitan air pollution. Their findings show that increased per capita GDP will lead to various investments that benefit the environment. [45], evaluate the strength of the association among income and carbon emissions in Turkey. The authors discovered a correlation between rising CO2 emissions and rising income. The authors find that the underlying premises of the EKC are not met. To put EKC to the test [46], investigated the correlation between CO2 and GDP in 16 industrialized OECD nations. The authors confirm that, from 1970 to 1980, inverted U-shaped patterns can be seen in most nations considered. As the cubic model predicted, the entire data set has the shape of [47] discovered an N-shaped pattern between economic expansion and carbon dioxide emissions in Austria between 1960 and 1999 using the same cubic model. [48], Analyze the relationships between health spending, economic growth, and carbon emissions in 51 countries. The GMM and dynamic simultaneous equation linear models are employed in this process. The researchers show a positive feedback loop between GDP growth and healthcare spending. In G7 countries [49], explained contrasting opinions. Carbon emissions encourage clean energy use in Germany, but in the United States, Canada, and the United Kingdom, the data demonstrate that reliance on renewable energy sources enhances GDP. In addition, the analysis finds a unidirectional correlation between renewable energy consumption and CO2 emissions in the United States while finding bidirectional causality between these two variables in Germany. [50], provide additional evidence that investment and economic growth contribute to rising CO2 emissions in the United States. However [51], examines the impact of GDP square on environmental degradation in Southern European countries from 1990 to 2018 controlling economic growth (CEG), globalization, and energy consumption (EC). Results show that an increase in GDP square leads to an increase in CO2 emissions, while an increase in economic growth boosts emissions. This information is crucial for policy decision-making and mitigation efforts. According to research [52], a rise in carbon emissions has been associated with higher incomes and increased energy use in the United States. Furthermore [53], report that the main variables driving France's carbon emissions growth rate are energy consumption and economic growth. The study concluded that economic development in Malaysia did not affect the rate of increase in energy consumption. [54], investigates the potential correlations between income, energy consumption (EC) and the increase in Tunisia's CO2 emissions. According to the study, increased carbon emissions result from increased energy consumption, stimulating economic growth. A multivariate technique was used to investigate the relationships between China's carbon emissions, economic growth, and energy use [55] used a multivariate technique. Economic expansion is shown to have a beneficial effect through increasing energy consumption, whereas the usage of energy from vestige fuels is shown to accelerate the increase in carbon emissions. Bangladesh's CO2 emissions, ED and EC are all evaluated by Ref. [56]. The vector error correction Model, (VECM) shows that carbon emissions and energy use are going in both directions. The estimators also show that the variables being studied are long-term cointegrated. In addition, a unidirectional trend verifies the transformation from carbon emissions to economic expansion. Energy intensity, GDP, and carbon emissions in Greece are all linked in a VECM study [57]. According to the VECM calculations and Johansen's multivariate cointegration, economic expansion increases energy intensity and carbon emission levels. A feedback loop between energy use and carbon output was also discovered.

[58] run their model on CO2 emissions using data from 1995 to 2016. Their findings corroborate the idea that rising demand for fossil fuels is a driving factor in China's rising carbon output. According to Ref. [59], rising carbon emissions in ASEAN result from increasing economic output and energy use. To analyze the connection between GDP growth, energy usage, and carbon emissions [60], used a comparable panel data set of 116 nations. According to the author's research, nations in the North Africa and Middle East (MENA) area have higher carbon emission growth rates due to their nonrenewable energy sources than countries in sub-Saharan Africa, the Caribbean, and Latin America. Results show that carbon emissions do not affect energy consumption for most nations worldwide. Results were comparable in the case of Italy from 1970 to 2014, as demonstrated by Saint [11].

## 3 Methodology

### 3.1 Conceptual framework

**Fig. 1:**
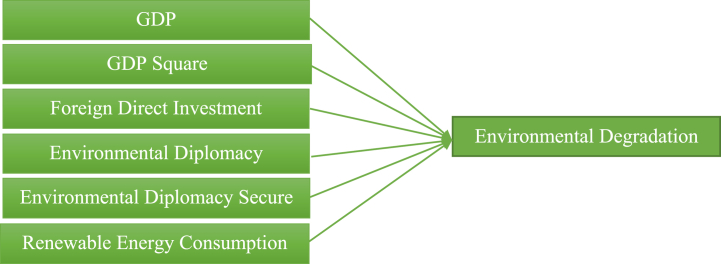
Conceptual framework.

A conceptual framework provides a graphical representation of the expected relationship among variables. The research process is defined by developing significant objectives and a systematic incorporation of these objectives to derive coherent and cohesive results. The expected cause, “GDP, GDP square, foreign direct investment, environmental diplomacy, environmental diplomacy secure, and renewable energy,” are the independent variable (predictor or explanatory variable). Environmental degradation is the response or outcome variable that is the expected effect of analysis. The entire framework of the investigation is depicted in Fig. 1.

### 3.2 Hypothesis development

H1 The GDP has a significant impact on environmental degradation.

H2 The GDP square has a negative and significant impact on environmental degradation.

H3 Foreign Direct Investment has a significant impact on environmental degradation.

H4 Environmental Diplomacy has a significant impact on environmental degradation.

H5 Environmental Diplomacy Secure has a significant impact on environmental degradation.

H6 Renewable Energy Consumption has a significant impact on environmental degradation.

### 3.3 Data

Examining the effects of variables (GDP, GDP square, foreign direct investment, environmental diplomacy, environmental diplomacy secure, and renewable energy) on environmental degradation (CO2 emissions) in OECD countries, the study employs eviews-10 and panel data gathered from the World bank and WDI between 1991 and 2020. The analysis findings will help clarify the relationship between environmental degradation in OECD countries over the given period of time and various factors, including GDP, GDP square, foreign direct investment, environmental diplomacy, and secure and renewable energy consumption.

### 3.4 Model speciﬁcation

Several factors, including GDP, GDP square, FDI, environmental diplomacy, security, and renewable energy consumption, may significantly impact CO2 emissions. Sustainable development can directly influence the motivation of governments, businesses, and individuals to reduce carbon emissions. We can reduce carbon emissions if firms and households follow environmental policies more strictly. Through increased economic activity, sustainable development may indirectly increase energy consumption. Governments may seek measures that increase carbon dioxide emissions if environmental policy needs to be clarified due to poor economic growth [61]. Firms with large carbon footprints are departing the industrialized world for regulatory certainty [62].

While rapid economic expansion can cause environmental degradation, environmental legislation can address public concerns while correcting market failings that exacerbate the problem [63]. Reducing pollution is vital to limit overall energy consumption and create incentives for energy efficiency [64]. The empirical model for reducing CO2 emissions considers elements such as GDP, GDP square, foreign direct investment, environmental diplomacy, environmental diplomacy security, and renewable energy consumption. This study adheres to the following model as shown in equation (1):

(1) $$\mathrm{Ln}\left({Co2}_{t-1}\right)=\beta_{0}+\beta_{1}\;\mathrm{Ln}\left({GDP}_{t-1}\right)+\beta_{2}\;\mathrm{Ln}\left({GDP}_{t-1}^{2}\right)+\beta_{3}\;\mathrm{Ln}\left({FDI}_{t-1}\right)+\beta_{4}\;\mathrm{Ln}\left({ED}_{t-1}\right)+\beta_{5}\;\mathrm{Ln}\left({EDS}_{t-1}\right)+\beta_{6}\;\mathrm{Ln}\left({REC}_{t-1}\right)+{\mu \;}_{t-1}$$

Table 1 describes the variables used in the model.

**Table 1:** Description of variable.

Variable	Code	Measurement
Gross Domestic Product	*GDP*	GDP growth rate (% of annual GDP growth rate)
Gross Domestic Product square	GDP2	GDP growth rate square root (Square root of % of annual growth rate of gross domestic product)
Foreign Direct Investment	*FDI*	Net inflows (% of GDP)
Environmental Diplomacy	*ED*	Cumulative number of treaties
Environmental Diplomacy secure	*EDS*	Total, % of primary energy supply
Renewable Energy Consumption	*REC*	% of total final energy consumption
CO2 Emissions	*CO2*	Measured in metric tons per capita Carbon dioxide

### 3.5 Analysis techniques

[65], devised a test to examine cross-sectional dependence among panel elements. Following that, we apply the co-integration test, which delivers stable finding under heterogeneity and CD, to analyze the stability of the long term relationship between our research variables. This investigation engaged an updated version of the standard group mean estimate for related effects [66], consistent with recent findings.

We use a panel of FMOLS models for long-term estimates. FMOLS is one of the most important tools for estimating heterogeneity in panel econometrics [67]. This approach uses the cross-section estimates' long-run covariance and reweights the data to consider heterogeneity's impact on estimation. We employ the Group-FMOLS technique to forecast the medium- and long-term relationships between the parameters, which is why this approach is so significant. The PMG-ARDL model described in Ref. [68] was used to investigate the impact of sustainable development on CO2 emissions. Because of the pooled mean group, long-run coefficients must be consistent across countries, even if short-run dynamics differ greatly. Under the careful eye of the MG, coefficients can exhibit both short- and long-term heterogeneity. The PMG estimator exhibits superior long-term homogeneity than the Mean Group estimator, according to Ref. [69]. According to Pesaran et al. (1999), the ARDL is appropriate when the series integration order is single or mixed. This approach promotes consistent and precise estimation by eliminating endogeneity. According to Choi (2006), Bretung & Pesarans (2007); and Sajid et al. (2023), the PMG estimate exhibits more substantial long-term homogeneity than the estimation of the group means. This scenario employs the following empirical model as shown in equation (2):

(2) $${\Delta \mathrm{Ln}(Co2}_{t)=}\beta_{0}+\beta_{1}L_{n}\left({GDP}_{t-1}\right)+\beta_{2}\;\mathrm{Ln}\left({GDP}_{t-1}^{2}\right)+\beta_{3}\;\mathrm{Ln}\left({FDI}_{t-1}\right)+\beta_{4}\;\mathrm{Ln}\left({ED}_{t-1}\right)+\beta_{5}\;\mathrm{Ln}\left({EDS}_{t-1}\right)+\sum_{i=1}^{p}\alpha_{1}\Delta \mathrm{Ln}\left({GDP}_{t-1}\right)+\sum_{i=1}^{s}\alpha_{2}\Delta \mathrm{Ln}\left({GDP}_{t-1}^{2}\right)+\sum_{i=1}^{v}\alpha_{3}\Delta \mathrm{Ln}\left({FDI}_{t-1}\right)+\sum_{i=1}^{y}\alpha_{4}\Delta \mathrm{Ln}\left({ED}_{t-1}\right)+\sum_{i=1}^{w}\alpha_{5}\Delta \mathrm{Ln}\left({EDS}_{t-1}\right)+{ECT}_{t-1}+\epsilon_{t-1}$$

## 4 Results and findings/discussion

### 4.1 Descriptive statistics

The findings of descriptive statistics are presented in Table 2. This table presents the average values for the following indicators in the OECD countries: gross domestic product, gross domestic product square, foreign direct investment, environmental diplomacy, environmental diplomacy secure, and renewable energy consumption: 1.948, 0.991, 1.948, 0.976, 2.230, 6.001 and 2.419 respectively. The minimum and maximum values are −0.056, −4.539, −0.056, −7.199, −1.273, 4.852, and −0.821, respectively, and maximum values are 3.243, 3.193, 3.243, 5.457, 4.497, 6.507 and 4.354 respectively. The standard deviation values are 0.610, 0.799, 0.610, 1.249, 1.136, 0.411 and 1.053 respectively.

**Table 2:** Result of descriptive statistics.

	Ln (CO2)	Ln (GDP)	Ln (GDP^)	Ln (FDI)	Ln (EDS)	Ln (EDS)	Ln (REC)
**Mean**	1.948	0.991	1.948	0.976	2.230	6.001	2.419
**Median**	2.053	1.091	2.053	1.052	2.293	6.080	2.509
**Maximum**	3.243	3.193	3.243	5.457	4.497	6.507	4.354
**Minimum**	−0.056	−4.539	−0.056	−7.199	−1.273	4.852	−0.821
**Std. Dev.**	0.610	0.799	0.610	1.249	1.136	0.411	1.053

### 4.2 Correlation analysis

A correlation matrix shows the correlation among several factors and CO2 emission in OECD countries. According to the results, all factors positively and negatively correlated with CO2 emission. The estimated findings of the correlation matrix are shown in Table 3.

**Table 3:** Correlation analysis.

	Ln (CO2)	Ln (GDP)	Ln (GDP^)	Ln (FDI)	Ln (EDS)	Ln (EDS)	Ln (REC)
**Ln_CO2**	1						
	−1						
	–						
**Ln_GDP**	−0.100	1.000					
	−2.941	–					
	0.003	–					
**Ln_GDPS^**	1.000	−0.100	1.000				
	–	−2.941	–				
	–	0.003	–				
**Ln_FDI**	−0.014	0.155	−0.014	1.000			
	−0.416	4.589	−0.416	–			
	0.677	0.000	0.677	–			
**Ln_ED**	−0.451	0.022	−0.451	0.091	1.000		
	−14.74	0.644	−14.741	2.666	–		
	0.000	0.520	0.000	0.008	–		
**Ln_EDS**	−0.027	−0.049	−0.027	0.263	0.223	1.000	
	−0.800	−1.432	−0.800	7.966	6.671	–	
	0.424	0.153	0.424	0.000	0.000	–	
**Ln_REC**	−0.454	−0.002	−0.454	0.076	0.880	0.234	1.000
	−14.88	−0.069	−14.88	2.230	54.03	7.033	–
	0.000	0.945	0.000	0.026	0.000	0.000	–

### 4.3 Cross sectional dependence (CD) test and CPIS (Unit root test.)

The empirical section begins with Table 4 and shows the findings of a cross-sectional dependencies (CD) test using Psarian's (2004) technique. The findings point to cross-national dependencies and argue against accepting the null hypothesis of cross-sectional independence. This demonstrates that a disturbance in one country has indirect effects on the other countries in the study [27]. The CD test results provide the foundation for the next stage to examine the degree of series stationarity. To avoid inconsistencies, it is essential to consider that all research processes are independent. The CIPS employs the 2nd generation panel unit root test to confirm cross-national correlations. Table 4 displays the results of the unit root tests performed on the CIPS panel. There are numerous methods for determining whether something is stationary. As a result, the research engaged CIPS and other second generation unit root testing. We tested for stable variables using the constant alone and constant plus trend specifications and the level and first differences tests for unit roots, as shown in Table 4. A CIPS test confirmed that all variables of study are stationary. Even though two of the variables are not level, they all become stationary at the 1 % and 5 % difference and trend levels. I (1) demonstrate that the variables do not exhibit stationary behavior by integrating them into order one. This demonstrates how critical it is to perform a cointegration test as part of an ARDL-bound analysis to determine the long-term link between the variables. The unit root of the variable represents the value under this null hypothesis.

**Table 4:** Finding of CD and CIPS test.

Test	Ln (GDP)	Ln (GDP^)	Ln (FDI)	Ln (EDS)	Ln (EDS)	Ln (REC)
**CD test**	7.43	11.47	13.45	10.29	5.56	4.90
**CIPS (at level)**	0.007	0.008	0.000	0.004	0.000	0.003
	0.475 n0	0.509 n0	0.694 n0	0.753 n0	0.520 n0	0.499 n0
**CIPS (at 1st difference)**	0.032	0.044	0.039	0.035	0.054	0.024
	0.000 ***	0.000 ***	0.000 ***	0.000 ***	0.000 ***	0.000 ***

### 4.4 DOLS and FMOLS tests

Table 5 present the estimated values of FMOLS and DOLS. The impacts over time can be calculated using the coefficients provided by these models. For GDP^, FDI, and renewable energy utilization, respectively, its coefficient in the FMOLS and DOLS models is statistically significant at 5 % and 1 %. However, this yields positive and statistically significant outcomes for the gross domestic product square, environmental diplomacy, and environmental diplomacy square coefficient in the FMOLS and DOLS models at the 1 % and 5 % levels. FMOLS and DOLS are used to validate the long-term symbol and significance estimation of the ARDL model.

**Table 5:** FMLOS and DOLS test.

Test		Ln_GDP	Ln_GDP^2^	Ln_FDI	Ln_EDS	Ln_EDS	Ln_REC
**FMOLS**	Coefficient	0.011	−0.136	−0.016	0.023	0.000	−0.208
	t-Statistic	2.922	−3.009	−3.281	2.874	3.943	−8.211
	Prob.	0.004 ***	0.003 ***	0.001 ***	0.070 **	0.044 **	0.000 ***
**DOLS**	Coefficient	0.029	−0.277	−0.013	0.175	0.043	−0.209
	t-Statistic	2.593	−2.409	−2998	3.136	3.732	−4.989
	Prob.	0.010 ***	0.017 **	0.007 ***	0.004 ***	0.031 **	0.000 ***

### 4.5 PMG-ARDL test

We found that the PMG estimator is superior to the MG estimator when evaluating alternatives to H0. Table 6, Table 7 displays the output of PMG-ARDL. The PMG-ARDL examined the impact of factors such as gross domestic product; gross domestic product square, foreign direct investment (FDI), environmental diplomacy, and environmental diplomacy secure negatively and positively and significantly reason of carbon dioxide emissions in the long run in the OECD countries.

**Table 6:** Short-run impact.

Variable	Coefficient	Std. Error	t-Statistic	Prob. *
**D (Gross Domestic Product (-1))**	0.631	0.012	54.095	0.000***
**D (Gross Domestic Product Square)**	−0.024	0.001	−45.230	0.000***
**D (Foreign Direct Investment (-2))**	−0.004	0.001	−5.278	0.013***
**D (Environmental Diplomacy)**	0.012	0.001	14.824	0.001***
**D (Environmental diplomacy secure)**	0.179	0.014	12.695	0.001***
**D (Renewable Energy Consumptions)**	1.190	0.075	15.788	0.001***

(*) Significance level at 10 %,(**) Significance level at 5 % (***), Significant level at 1 %,(no) Not significant.

**Table 7:** Long-run impact.

Variable	Coefficient	Std. Error	t-Statistic	Prob.*
**Gross Domestic Product**	0.270	0.053	5.098	0.000***
**Gross Domestic Product Square**	−0.004	0.004	−1.130	0.259 no
**Foreign Direct Investment**	−0.144	0.016	−9.241	0.000***
**Environmental Diplomacy**	0.045	0.014	3.124	0.002***
**Environmental diplomacy secure**	0.004	0.000	10.988	0.000***
**Renewable Energy Consumptions**	−0.036	0.015	−2.433	0.015**

#### 4.5.1 PMG-ARDL short-run impact

The short-run active parameter of the PMG-ARDL Framework is thus real, and a model for short-run cointegration is born. The short-term results shows in Table 6. Significant level at 1 %, the short-term impact of GDP on CO2 emissions is significant (Coefficient = −0.631, t-value = 54.094, and p < 0.01). According to Ref. [70], gross domestic product has a short-run impact on CO2 emissions in line with results of the current investigation. Despite 1 % significance, the square of GDP has a significant and immediate influence on CO2 emissions (Coefficient = −0.024, t-value = −45.230, p < 0.01). According to Ref. [71], The current research demonstrates that GDP square affects CO2 emissions in the short-run. The short-term effect of FDI on CO2 emissions is detectable at the 5 % significance level (coefficient = −0.004, t-value = −5.278, and p < 0.05). According to Ref. [72], the conclusion of the current study, foreign direct investment affects CO2 emissions in the short run. At the 1 % level, there is a statistically significant association between environmental diplomacy and CO2 emissions (coefficient = 0.012, t = 14.824, and p < 0.01). This investigation's findings are consistent with those of [73], They contend that, in the short run, environmental diplomacy influences carbon dioxide emissions. Environmental diplomacy security has significant short term effect on CO2 emissions at 1 % significant level (Coefficient = −0.179, t-value = 12.695, p < 0.01). According to Ref. [74], According to the current study's findings, the effect of environmental diplomacy on CO2 emissions is only ephemeral. There is also statistical significance at the 1 % level in the short-term impacts of renewable energy on CO2 emissions (p 0 <0 .01, coefficient = 1.190 and t = 15.788, The findings of the current investigation are consistent with those of [75], which claims that using REC has short term impact on CO2 emissions.

#### 4.5.2 PMG- ADRL long-run impact

Table 7 shows the long-term outcome. GDP significantly affects CO2 emissions at the significant level 1 % (Coefficient = −0.270, t-value = 5.098, p-value = 0.000). According to Ref. [76], The current study demonstrates that GDP has a long-term impact on CO2 emissions. GDP has an insignificant long-term impact on CO2 emissions (Coefficient = −0.004, t-value = −1.130, and p-value = 0.259). In the long run, GDP squared does not affect carbon dioxide emissions. Foreign direct investment has a long-term significant influence on CO2 emissions at the 1 % level of significance (coefficient = −0.144, t-value = −9.241, and p-value = 0.000). According to Ref. [77], the findings of this study are reliable with the long-term influence of FDI on CO2 emissions. Environmental diplomacy (ED) has long-term effects on CO2 emissions at the 1 % level of significance (coefficient = 0.045, t = 3.124, and p-value = 0.002). This study revealed similar results to others. [78], According to which environmental diplomacy has a long-term impact on CO2 emissions. At the 1 % level of significance, an environmental diplomacy security policy will have a considerable influence on CO2 emissions in the short term (Coefficient = −0.004, t-value = 10.988, and p-value = 0.001). According to Ref. [79], The results of this analysis align with the idea that environmental diplomacy security affects CO2 emissions in the long run. Last but not least, at the 5 % level, the long-term effects of using renewable energy on CO2 emissions are statistically significant (coefficient = −0.036, t = −2.433, and p-value = 0.015). The results of this study are in line with those of [80], They claim that consumption of renewable energy affects carbon dioxide emissions in the long run.

### 4.6 Dumitrescu hurlin panel causality tests

This study also investigates how renewable energy consumption, environmental diplomacy, foreign direct investment, GDP, GDP square, and environmental diplomacy security affect CO2 emissions in OECD countries. Table 8 displays the finding of the causality test. There is a direct causal link between GDP*GDP and CO2. This evidence demonstrates that the GDP*GDP of OECD countries appropriately estimates the dangers of CO2 emissions. Furthermore, we discovered suggestion of a directional causal association between GDP and CO2, which suggests that changes in GDP will affect CO2 levels. We also discovered that FDI predicts CO2 in OECD countries, implying a unidirectional causal relationship between the two variables. Our discovery of a unidirectional causal link between ED and CO2 suggests that ED changes will considerably influence CO2. Furthermore, we identified indication of one way casual link between ED and CO2, implying that changes in ED will influence CO2. Our data indicate a one-way causal link between REC and CO2, implying that REC changes will influence CO2. Finally, the data leads to a sequential occurrence that can only occur in one direction. These facts are the foundation upon which OECD policymakers base their decisions.

**Table 8:** Dumitrescu hurlin panel causality Test.

Path of Causality	W-Stat.	Zbar-Stat.	Prob.
GDP^*GDP → Co2	8.807**	3.659	0.0439
Co2→GDP^*GDP	4.186	0.672	0.4589
**GDP → Co2**	7.732**	4.143	0.0163
**Co2 → GDP**	3.953	0.397	0.5921
**GDP^ → Co2**	6.539**	4.016	0.0347
**Co2 →GDP^**	3.346	0.791	0.6010
**FDI →Co2**	7.998**	3.170	0.0506
**Co2 → FDI**	3.995	0.716	0.4997
**ED →Co2**	4.970**	1.943	0.0299
**Co2 →ED**	4.013	0.599	0.4971
**EDS →Co2**	4.978**	2.080	0.0396
**Co2 →EDS**	2.504	0.925	0.5549
**REC^ →Co2**	5.875*	1.980	0.0795
**Co2 →REC**	3.819	0.865	0.6012

**Notes:** Significance level at 10 % (*), Significance level at 5 % (**), Significance at 1 % (***).

### 4.7 Discussion

For GDP, FDI, and REC, the FOMLS and DOLS models reveal at 1 % negative and statistically significant coefficient. At 1 %, the GDP coefficient is positive and statistically significant. The FMOLS and DOLS models both contain positive coefficients for environmental diplomacy and its square. FMOLS and DOLS both validate the long-term importance and sign of the ARDL model. Because the PMG-ARDL Framework comprises both active parameters with short and long-term periods, we built a short-run cointegration model. The findings demonstrate the effects today and in the future: GDP has a significant impact on CO2 emissions both in the short and long run at the 1 % relevance level. According to (Chenran et al., 2019:Chaabouni & Saidi, 2017), The results of this research support earlier research suggests a short- and long-term link between GDP and CO2 emissions. GDP growth and CO2 emissions are mutually related. While GDP squared (GDP2) has a huge immediate influence on CO2 emissions, it has a minimal long-term effect (Co2). According to (G. Li et al., 2020; Baek, 2016), The current analysis supports prior findings that the square of GDP does not influence CO2 emissions in the short or long run. Foreign direct investment (FDI) substantially affects CO2 emissions in the medium and long run at the 1 % and 5 % significance levels, respectively. According to Tayebi et al. (2016); Alam et al. 2 (012), According to the current study's findings, Foreign direct investment impacts CO2 emissions in both the short and long term. The short- and long-term effects of environmental diplomacy (ED) on CO2 emissions have statistical significance at the 1 % level. The findings of this investigation are consistent with those of (Alam et al., 2012; Ang, 2008), According to research, environmental diplomacy can immediately and have a long-term impact on carbon dioxide emissions. At the 1 % significance level, EDS has a considerable immediate and long-term effect on CO2 emissions. According to [ [9,12,81]] The current study's findings support the idea that environmental diplomacy's security effects carbon dioxide emissions in the short and long run. An investigation concluded that the long-term and short-term effects of REC on CO2 emissions are statistically significant to a 1 % degree. The findings of the current investigation are consistent with those of (Wardhani & Dugis, 2020; Nwafor, 2014), which describe that REC has a short run and long run effect on CO2 emissions. Economic considerations and environmental diplomacy are closely intertwined and significantly impact environmental degradation. While economic pressures can positively or negatively impact environmental deterioration, environmental diplomacy allows states to collaborate to solve global environmental concerns. To achieve global sustainable development, finding a balance between economic development and environmental protection is critical. Governments, corporations, and civil society groups must all collaborate to achieve this goal.

## 5 Conclusion

In conclusion, the relationship between environmental diplomacy and economic concerns contributing to environmental deterioration has various elements. Mitigation must be a global, collaborative endeavor that addresses economic and environmental issues to be effective. To achieve this balance, it is vital to maintain diplomatic efforts, adopt new regulations, and commit as a community to environmental conservation. How GDP, GDP square, foreign direct investment, environmental diplomacy, environmental diplomacy secure, and renewable energy consumption affect Co2 emissions is the study's main objective. A complex relationship exists between GDP and CO2 emissions. New technologies, legislation, and energy sources can reverse this trend of rising emissions and a growing economy. Global challenges include sustainable economic development and carbon dioxide reduction. Foreign direct investment affects greenhouse gas emissions differently depending on the situation. By producing new technologies and adopting efficient procedures, FDI can strengthen environmental rules and cut emissions. FDI's excellent CO2 emission-cutting effect requires strong environmental policies and sustainable investment practices. Environmental diplomacy is essential to fighting global warming and carbon emissions. Negotiate, agree, and coordinate to develop policies, measures, and financial support to meet global climate targets and mitigate rising CO2 emissions. The regional energy mix, renewable energy deployment, legislation, and incentives to promote renewable energy adoption all affect renewable energy consumption and CO2 emissions. Growing evidence shows that switching to renewable energy sources is essential for decreasing CO2 emissions and fighting climate change.

### 5.1 Policy

This study suggests a comprehensive policy approach to address the association between economic growth and environmental degradation. It suggests decoupling economic growth from environmental impact, integrating FDI policies with environmental regulations, and promoting international collaboration. It also calls for a shift towards renewable energy consumption, focusing on sustainability, resilience, and global cooperation.

### 5.2 Implications

#### 5.2.1 Theoretical implications

This research could improve economic and environmental theory. The analysis can prove or invalidate the Environmental Kuznets Curve using OECD nations. This shows how economic growth harms the environment. The study can help strengthen environmental diplomacy theory by showing how international accords and diplomatic activities affect sustainability. It can aid international relations and environmental governance studies. The study also reveals that renewable energy protects the environment, supporting energy transformation. Foreign direct investment and CO2 emissions can help explain globalization and its environmental effects. Finally, regional variances in environmental diplomacy show how geography and geopolitics affect international environmental cooperation. This illuminates worldwide environmental governance.

#### 5.2.2 Practical implications

Findings have substantial implications for OECD officials and stakeholders. Actionable insights inform evidence-based economic and environmental strategies. The suggestions can assist governments in improving environmental diplomacy and international agreements. The research can also help regulate renewable energy to speed the transition to cleaner, more sustainable systems. Foreign direct investment regulations should also address the environment. This will enable host nations to attract green FDI. The paper encourages OECD countries to share best practices to reduce regional environmental diplomacy gaps and meet global climate and sustainability goals.

### 5.3 Limitations and future directions

In this study the relationship between environmental diplomacy, economic indicators, and CO2 emissions in OECD countries are discussed, although it has limitations. Data constraints and figure accuracy may reduce findings robustness. The study may only address part of the contextual factors that determine environmental outcomes, needing further investigation. Understanding OECD differences requires country-specific analyses. Future research initiatives may overcome these limitations by including finer-grained, regionally-specific investigations and using advanced econometric techniques to demonstrate causal relationships. Furthermore, because the study restricted itself to examining a few key variables, there is potential to examine other factors. Add air and water quality indicators to the research to better understand environmental sustainability. Future research should explore the dynamics of environmental diplomacy projects and technological innovation's impact on reducing environmental harm, thereby refining policy recommendations, and promoting a more sustainable global direction.

## Funding

This study was supported by Sichuan Provincial Department of Human Resources and Social Security 2023 research funds (Grant numbers. TB2023083).

## Data availability

The datasets used and analyzed during the current study are available from the corresponding author upon reasonable request.

## Declarations

**Ethics Approval and consent to participate:** This is an observational study. We confirmed that no ethical approval is required.

## Consent to participate is not applicable

**Consent for publication:** Not applicable.

## CRediT authorship contribution statement

**Mohammad Maruf Hasan:** Investigation, Funding acquisition, Formal analysis, Data curation, Conceptualization. **Su Nan:** Conceptualization, Project administration, Supervision, Writing - review & editing. **Muhammad Rizwanullah:** Formal analysis, Data curation, Conceptualization.

## Declaration of competing interest

The authors declare that they have no known competing financial interests or personal relationships that could have appeared to influence the work reported in this paper.
